# Supplementary material for: High-frequency repetitive transcranial magnetic stimulation protects against 6-OHDA-induced Parkinson’s disease symptoms by modulating the proNGF-p75NTR-sortilin pathway
Source: PeerJ. 2025 Jul 2;13:e19633. doi: 10.7717/peerj.19633 (PMC12228483; doi:10.7717/peerj.19633)
Supplement: Supplemental Information 2 [file peerj-13-19633-s002.docx]

STAT for Fig. 2 A


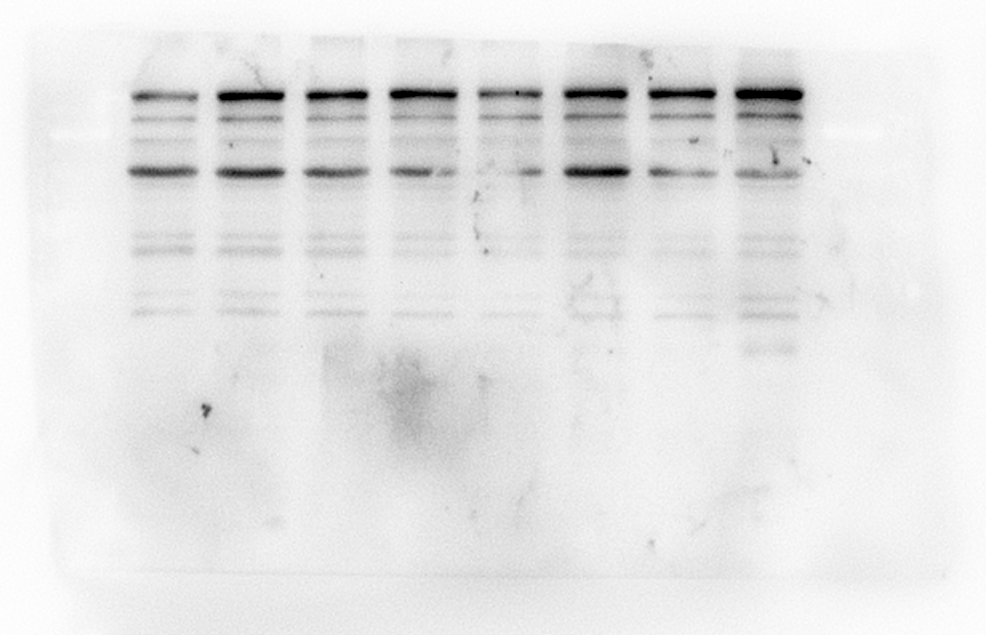

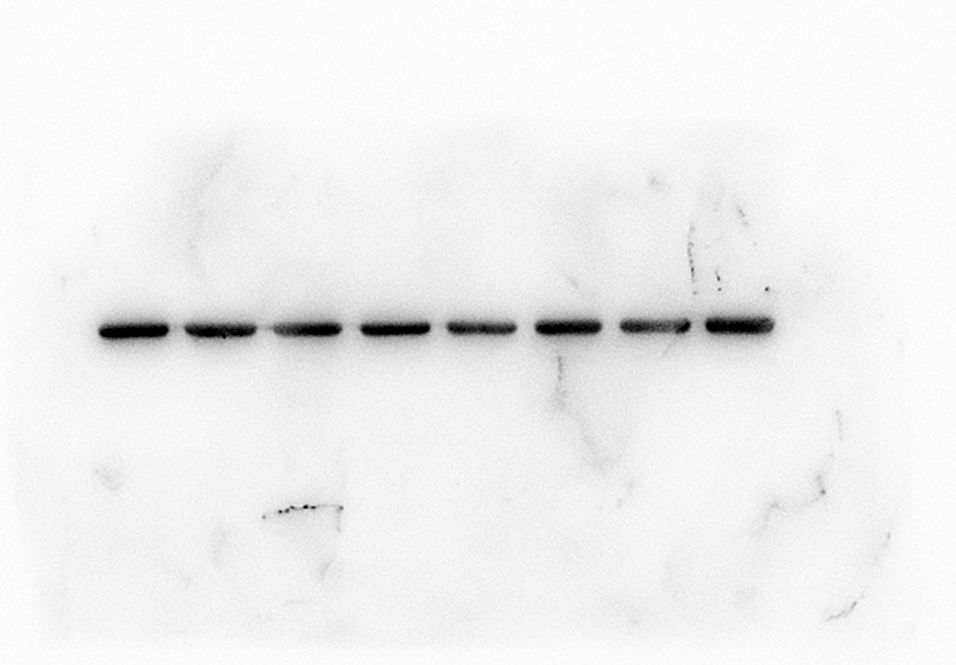


➀


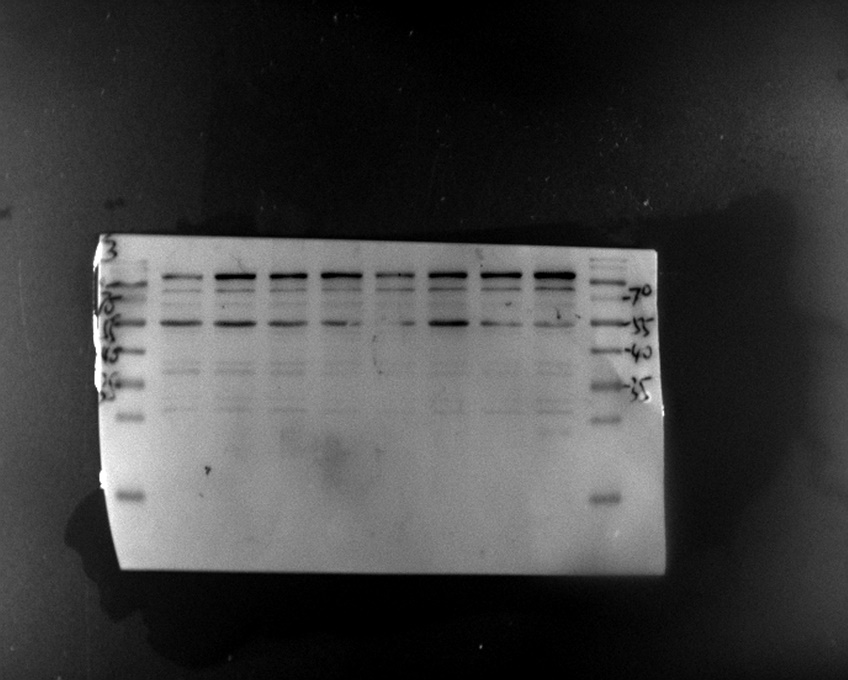

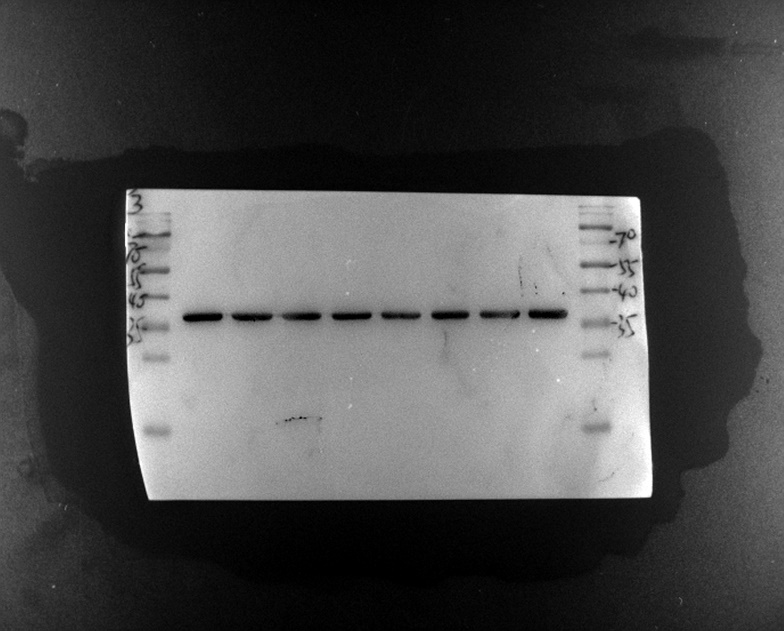


Sham-sortilin

Con

Ipsi

Ipsi

Con

Con

Ipsi

Ipsi

Con

GAPDH

STAT for Fig. 2 C


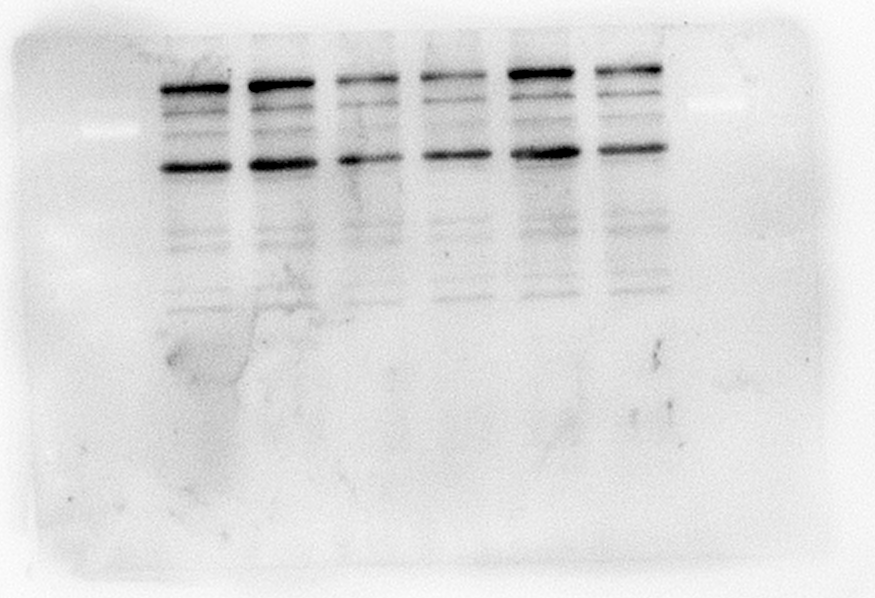

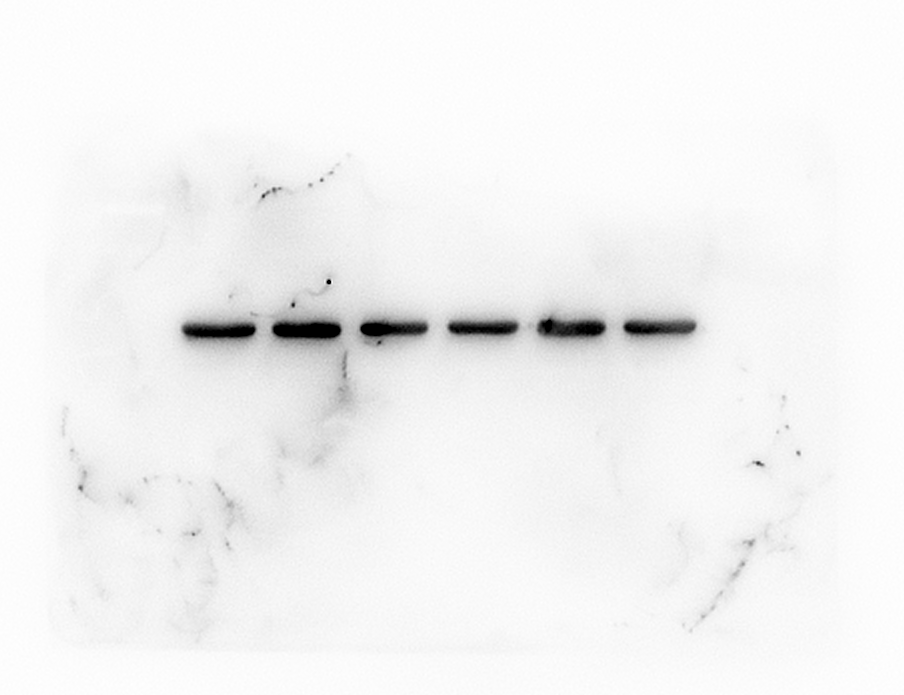


**➀**


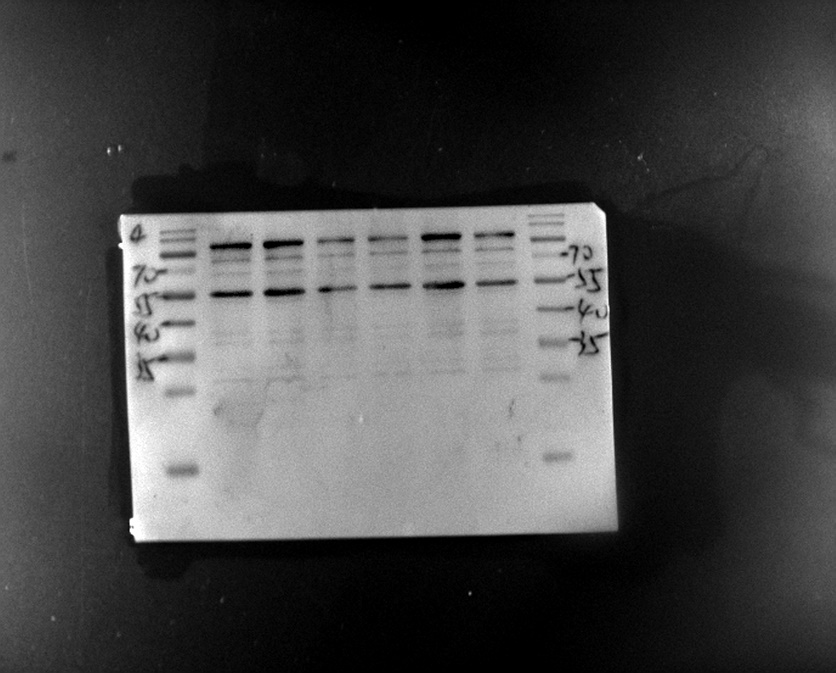

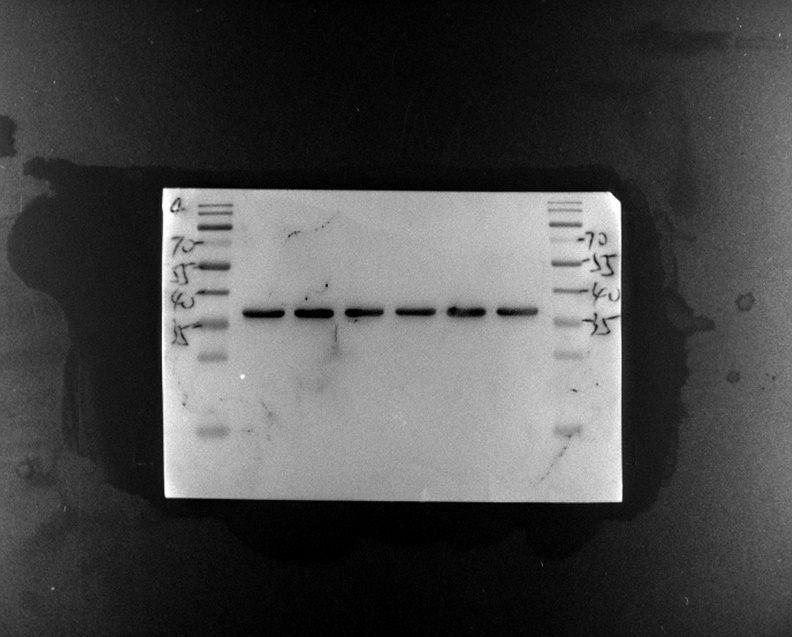


HF rTMS-sortilin

Ipsi

Con

Ipsi

Con

Ipsi

Con

GAPDH

Con

STAT for Fig. 3 A


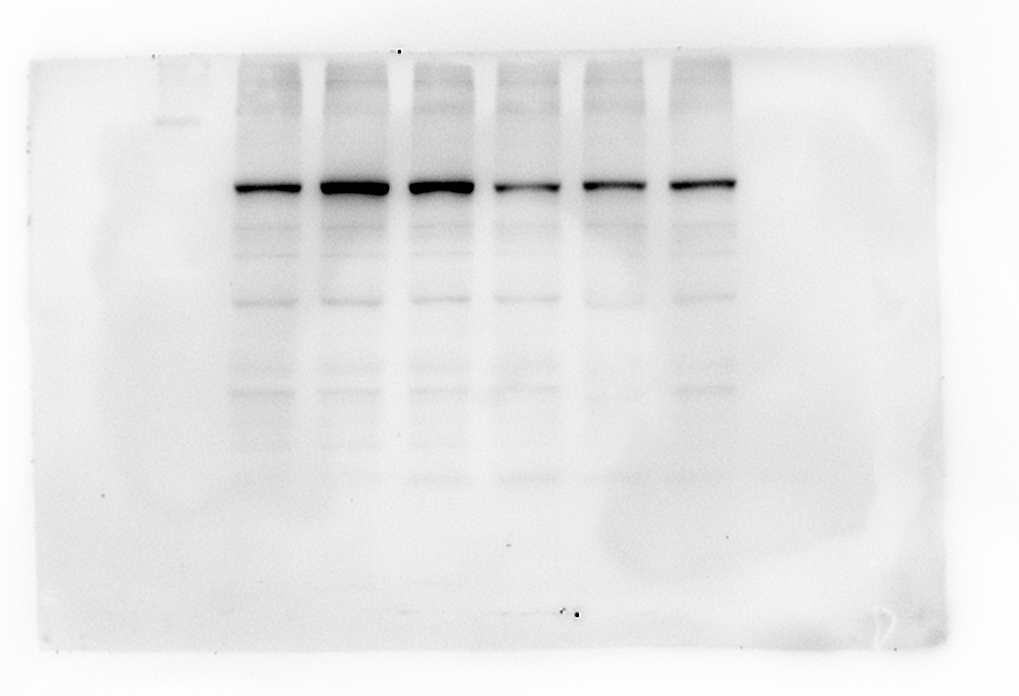

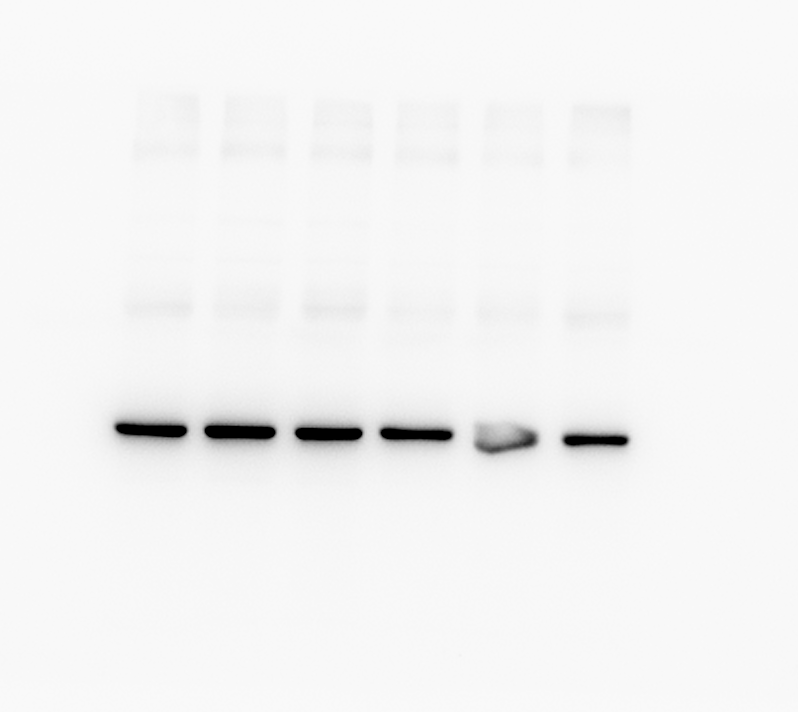


**
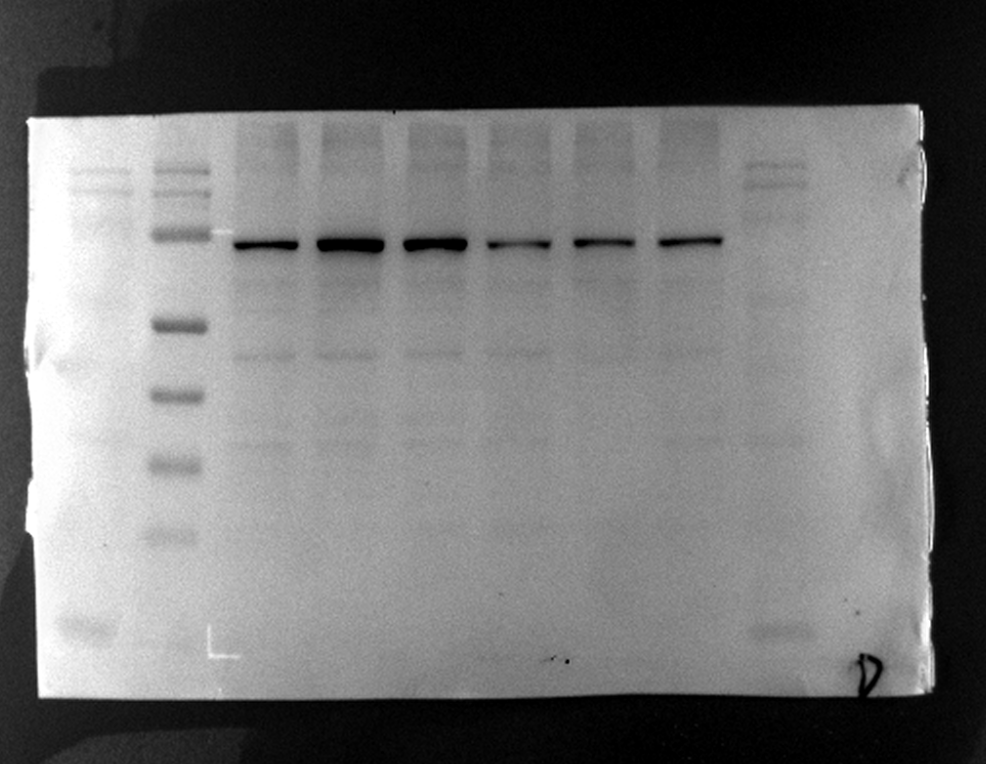
**
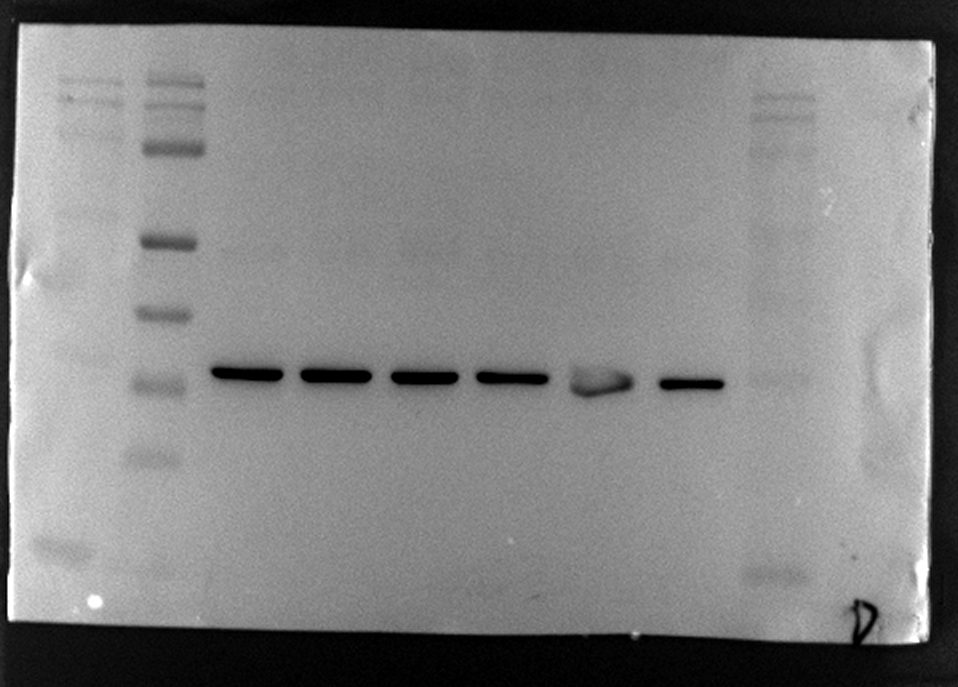


**➀**

**60**

**50**

**40**

**70**

**50**

**40**

**60**

**70**

GAPDH

Ipsi

Con

Ipsi

Con

Ipsi

Con

Sham-p75NTR

STAT for Fig. 3 C


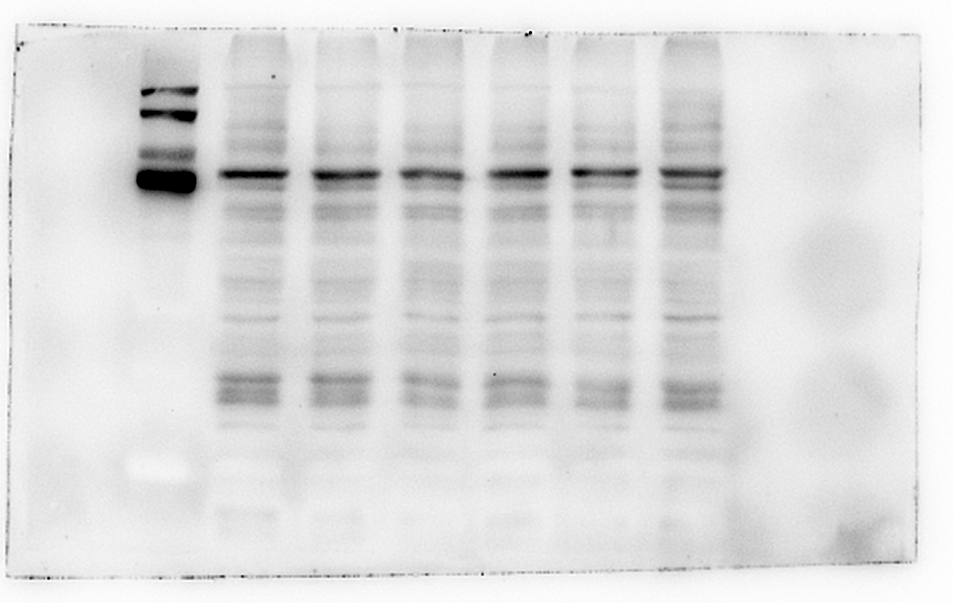

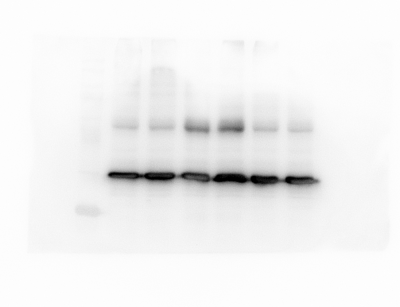


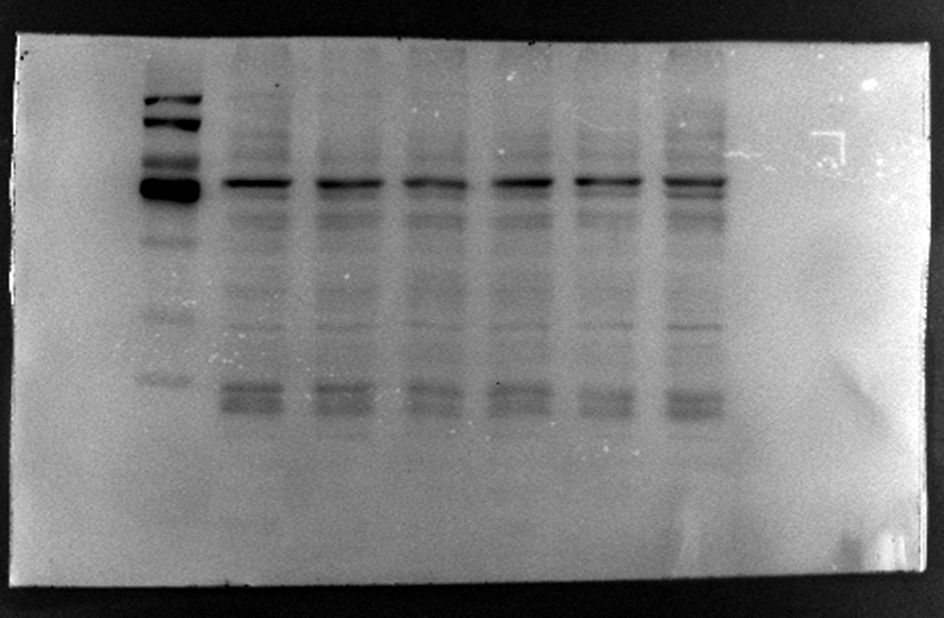

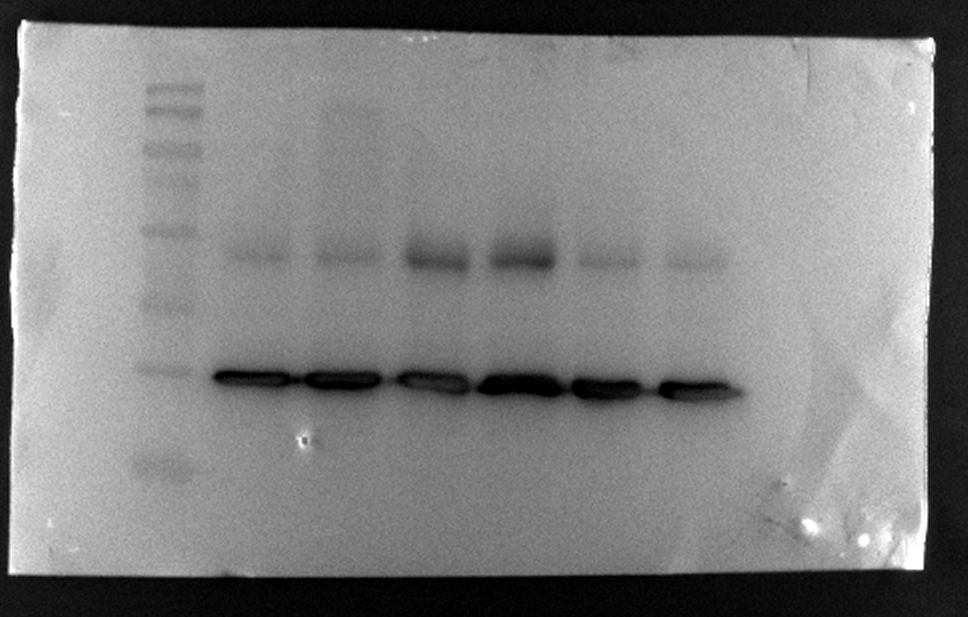


**➀**

**40**

**50**

**60**

**70**

**50**

**60**

**40**

**70**

GAPDH

HF rTMS-p75NTR

Con

Ipsi

Con

Ipsi

Ipsi

Con

GAPDH

Ipsi

Con

**40**

**70**

HF rTMS-p75NTR

STAT for Fig. 4 A


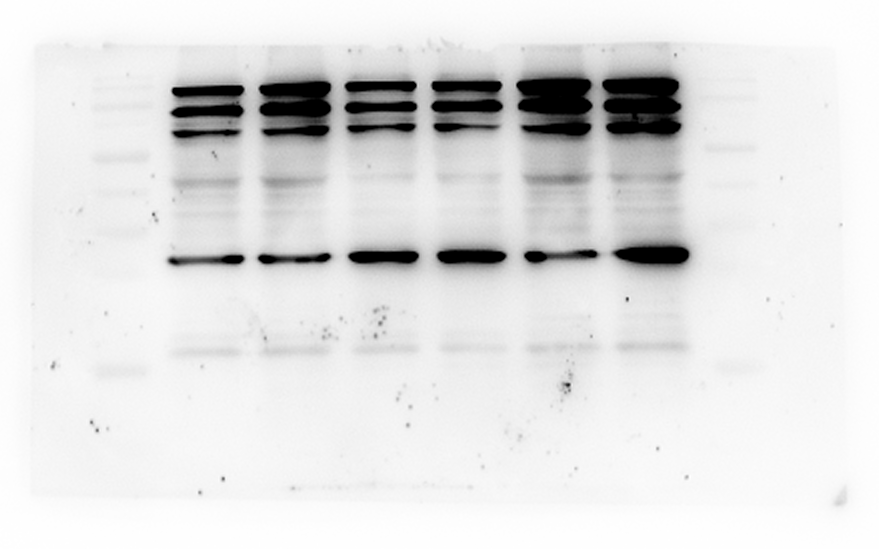

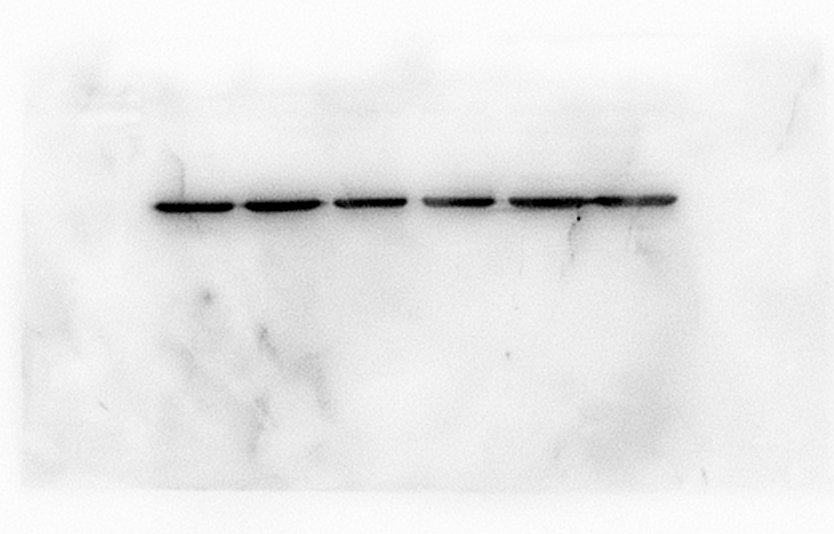


**➀**


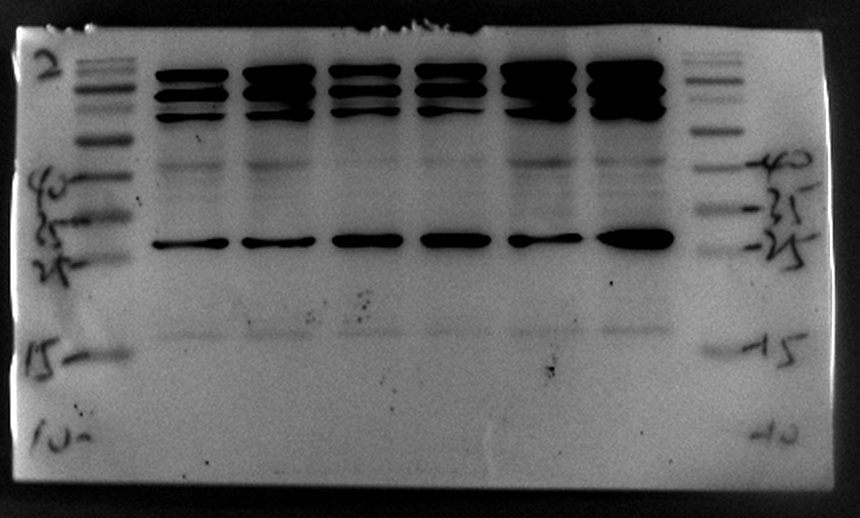

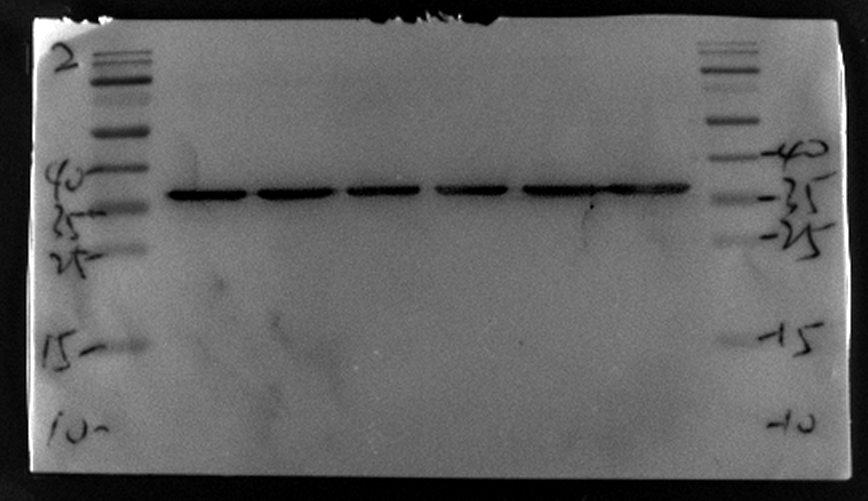


Ipsi
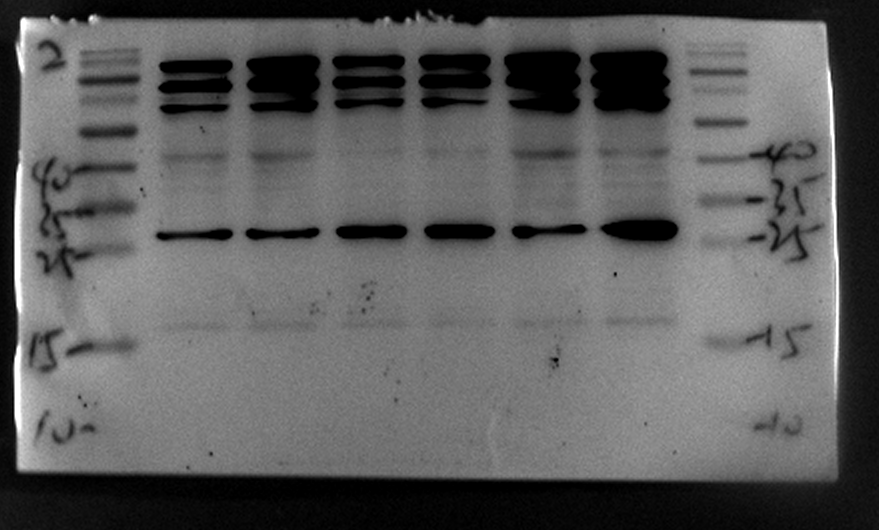


Con

Ipsi
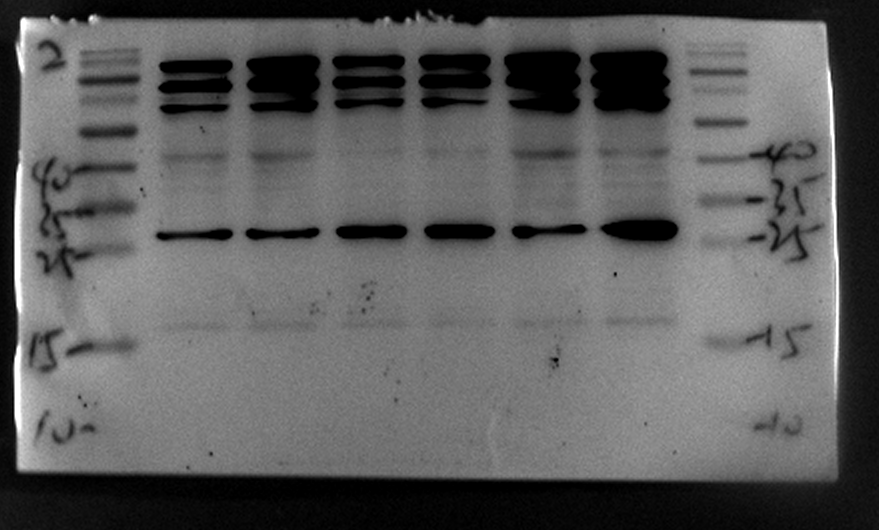


Con

Sham-pro NGF

GAPDH

Con

Ipsi
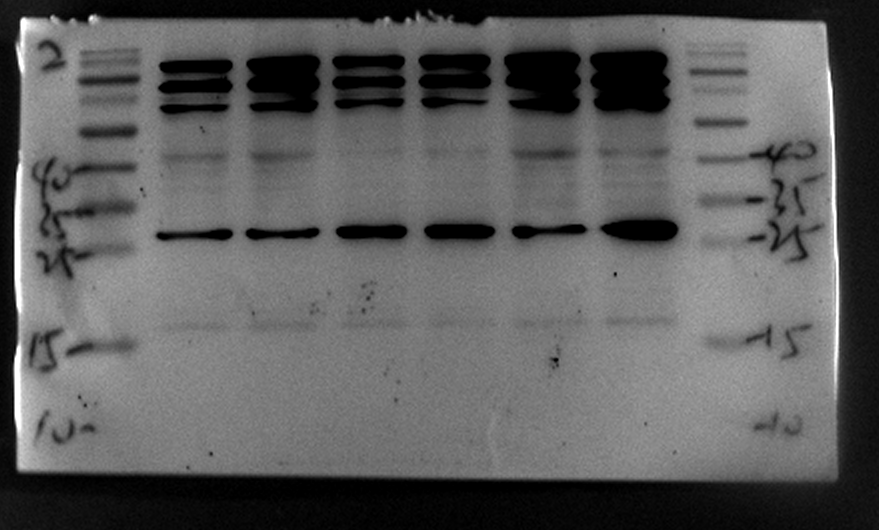


STAT for Fig. 4 C


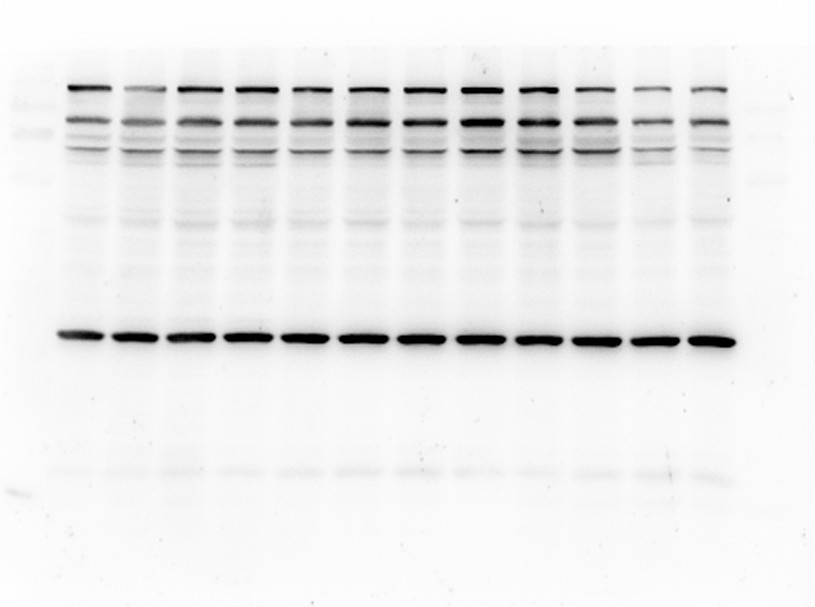

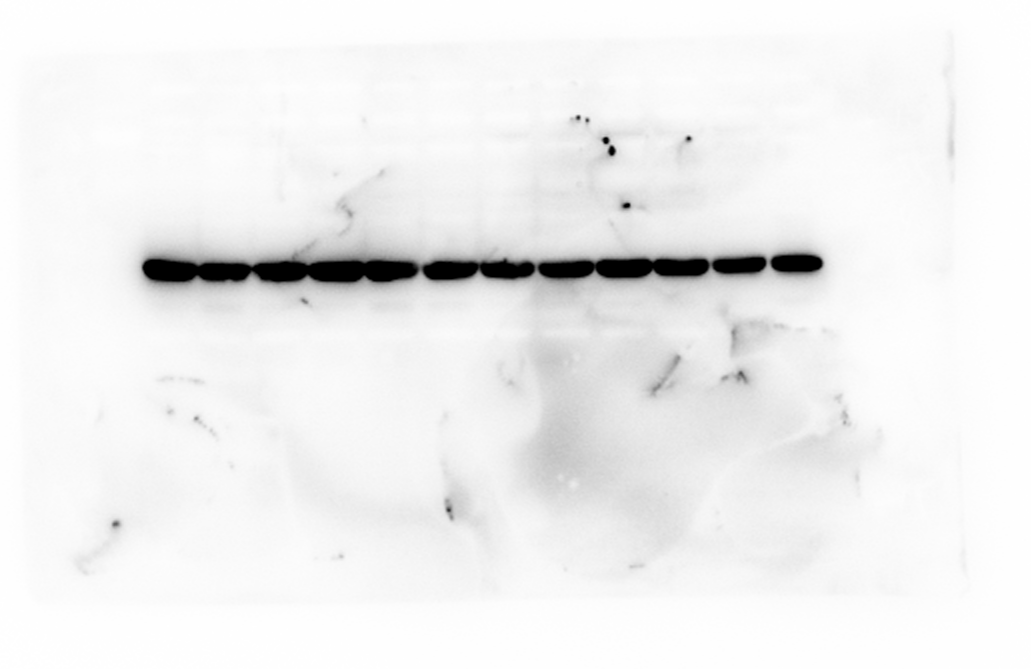


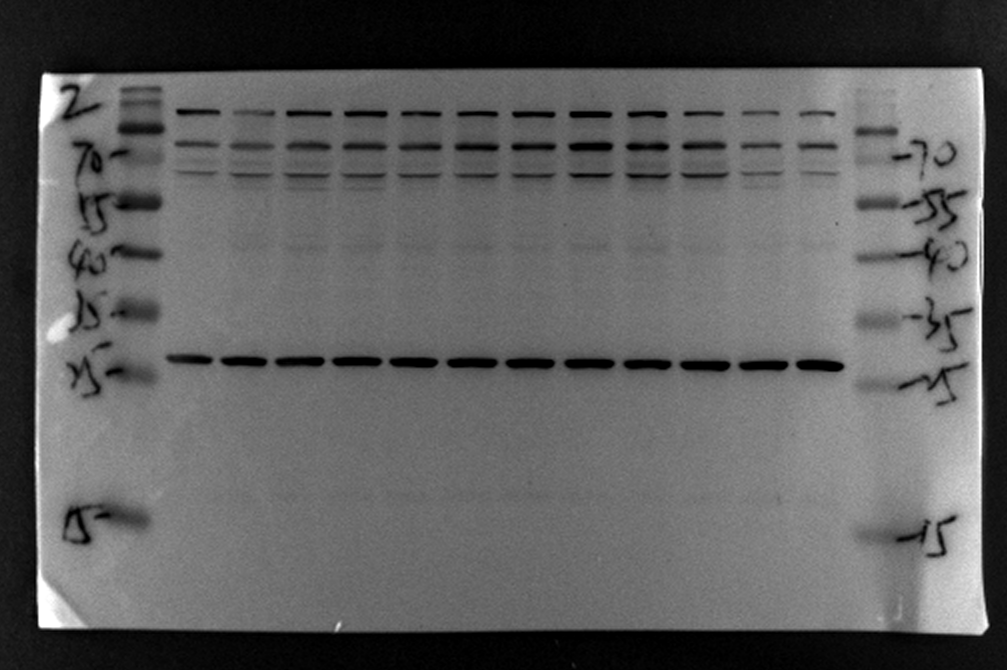

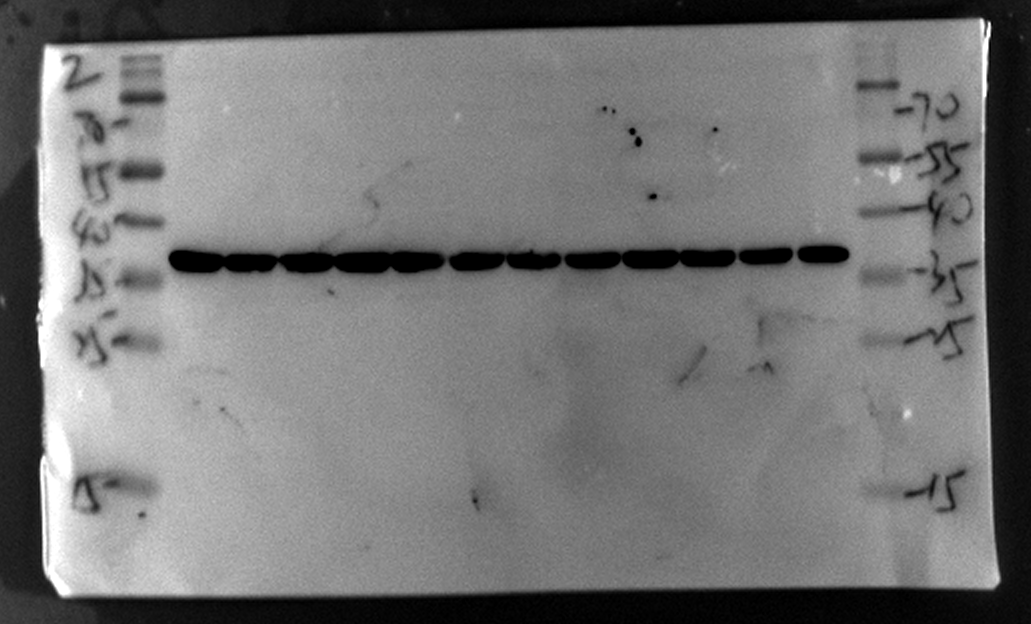


**➃**

**➀**

GAPDH

HF rTMS-pro NGF

Con

Con

Con

Con

Ipsi

Con

Ipsi

Ipsi

Con

Ipsi

Ipsi

Ipsi

STAT for Fig. 4 G


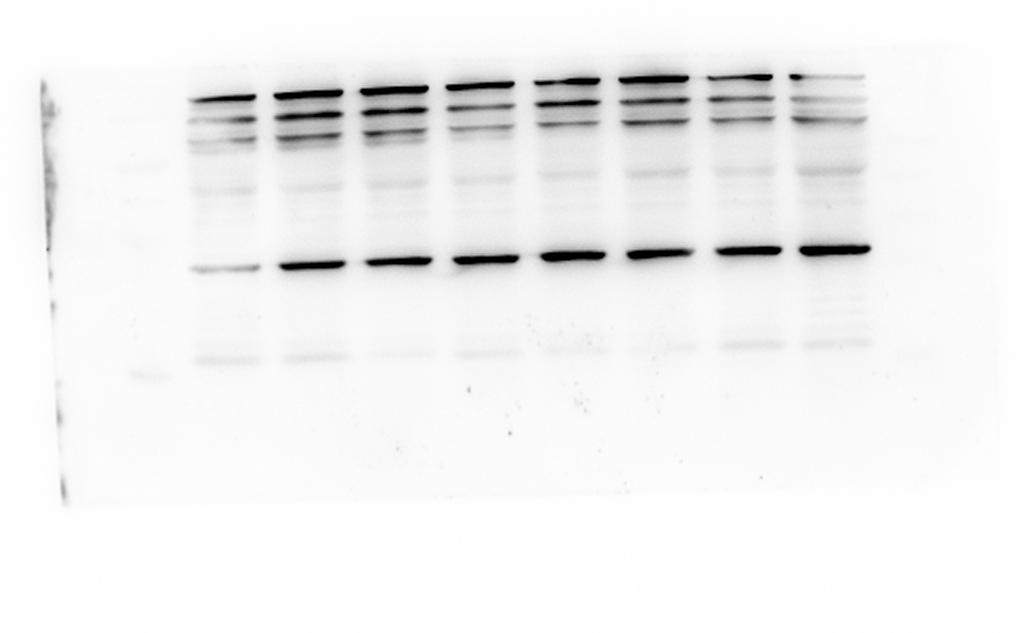

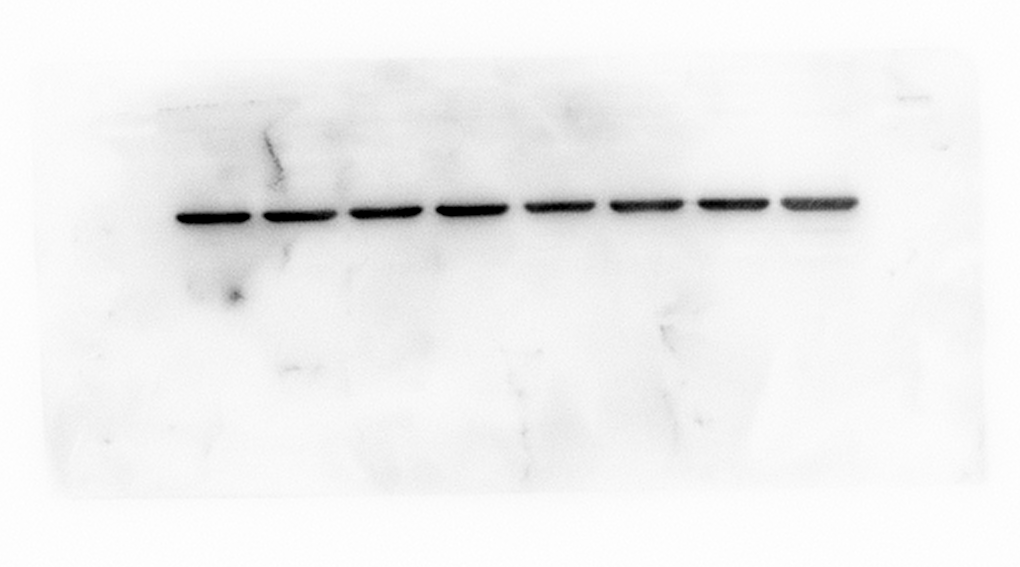


**➀**

**➃**


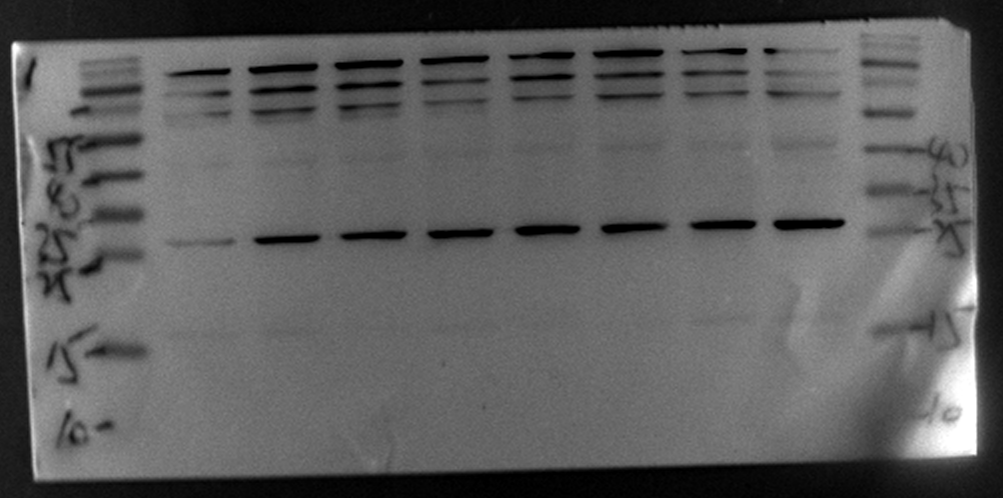

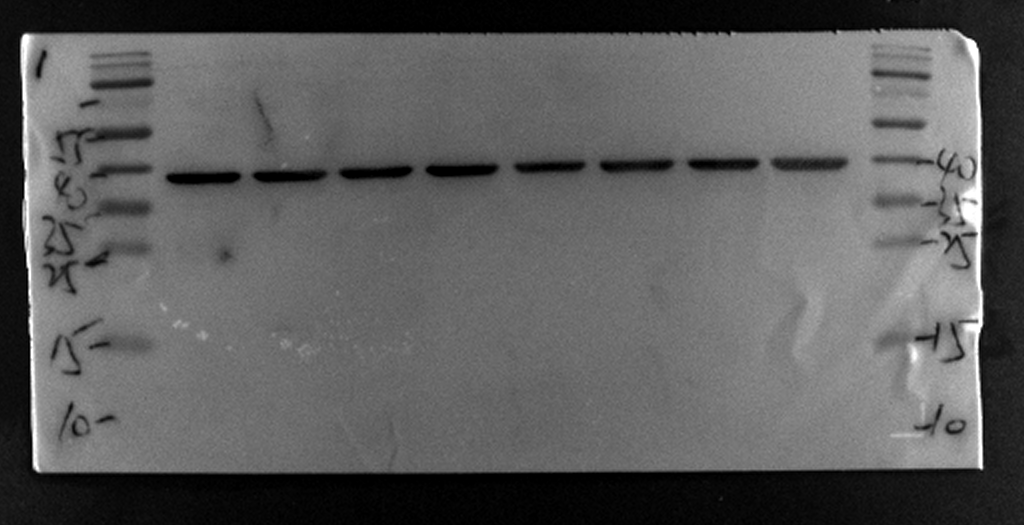


Sham-pro BDNF

Ipsi

Con

Ipsi

Con

Ipsi

Con

Ipsi

GAPDH

Con

STAT for Fig. 4 I


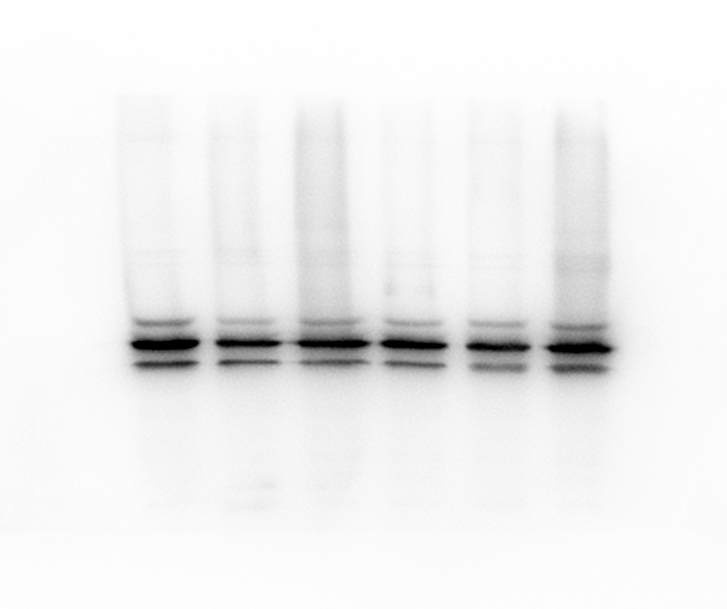

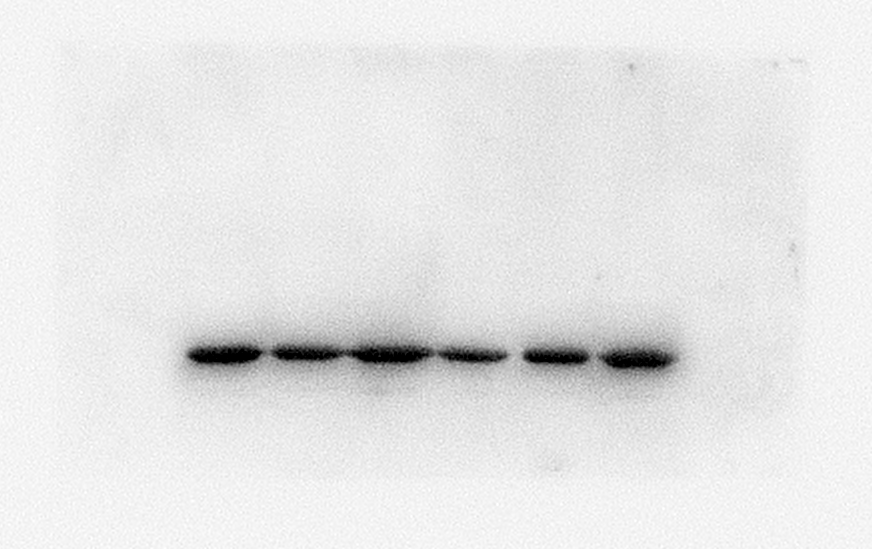


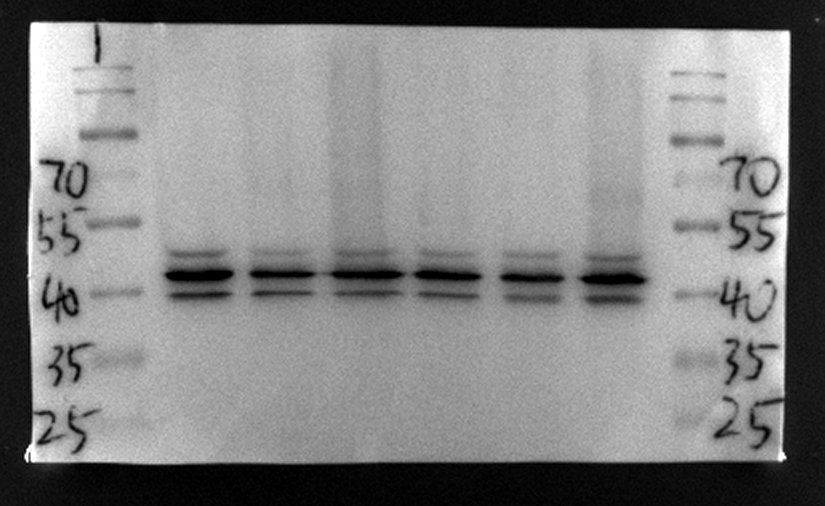

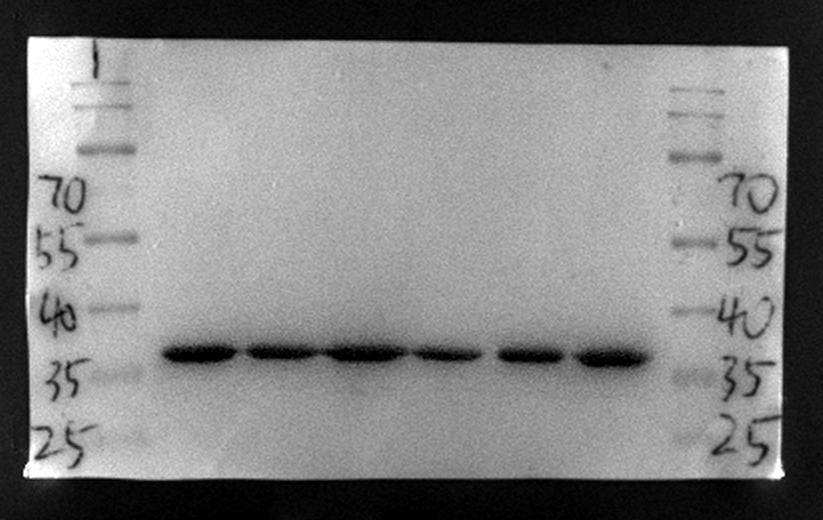


**➀**

GAPDH

HF rTMS-pro BDNF

Ipsi

Con

Con

Ipsi

Ipsi

Con

\
